# The role of pattern electroretinograms and optical coherence tomography angiography in the diagnosis of normal-tension glaucoma

**DOI:** 10.1038/s41598-021-91813-z

**Published:** 2021-06-10

**Authors:** Sang Yeop Lee, Nak-Hoon Son, Hyoung Won Bae, Gong Je Seong, Chan Yun Kim

**Affiliations:** 1 Department of Ophthalmology, Yongin Severance Hospital, Yonsei University College of Medicine, Yongin, Republic of Korea; 2 Department of Ophthalmology, Institute of Vision Research, Severance Hospital, Yonsei University College of Medicine, Seoul, Republic of Korea; 3 Data Science Team (Biostatistician), Center for Digital Health, Yongin Severance Hospital, Yonsei University College of Medicine, Yongin, Republic of Korea

**Keywords:** Optic nerve diseases, Glaucoma

## Abstract

In this study, we investigated the correlation between pattern electroretinogram (PERG) and optical coherence tomography angiography (OCTA) parameters for diagnosis in patients with normal-tension glaucoma (NTG). Forty-nine normal individuals (49 eyes) and 60 patients with NTG (60 eyes) were enrolled. OCTA and PERG parameters, such as macular vessel density (VD) and the amplitude of N35–P50 and P50–N95, were measured. Correlation analyses were performed between the parameters, and the area under the curve (AUC) was used to identify their diagnostic ability for NTG. Macular VD and the amplitude of N35–P50 and P50–N95 showed significant differences between the normal individuals and patients with NTG. Correlation between P50 and N95 amplitude and macular VD was significant in the normal and early glaucoma groups. Macular VD showed a higher AUC value (0.730) than that of P50–N95 amplitude (0.645) in the early glaucoma group. In the moderate to severe glaucoma group, the AUC value of the amplitude of P50–N95 (0.907) was higher than that of macular VD (0.876). The results indicate that PERG and OCTA parameters may identify glaucoma in its early stage, based on the severity of glaucomatous damage in patients with NTG.

## Introduction

Pattern electroretinogram (PERG) is a test for evaluating the electrical signals generated in the retina when a patterned stimulus of a certain luminance is applied. PERG can confirm macular retinal ganglion cell (RGC) dysfunction; therefore, it can be used for the diagnosis and prognosis of conditions with impaired function of the RGCs, such as glaucoma. In the past, several studies have demonstrated the utility of PERG in the diagnosis of glaucoma^1–6^. Further, its utility in identifying early alteration of RGC function in patients with glaucoma is supported by various previous studies, indicating changes in the parameters related to PERG before visual function changes^7–9^ and following reduction of intraocular pressure (IOP)^10^.

Optical coherence tomography angiography (OCTA), a recently developed imaging modality, is used for observing the microcirculation in the peripapillary or macular areas. Several studies have reported that in patients with glaucoma, OCTA may have a diagnostic ability comparable to that of optical coherence tomography (OCT)^11–13^. Moreover, some studies have identified a stronger association of OCTA with the visual function in comparison to OCT^14,15^. Further, according to earlier studies, the vessel density (VD) and flow index of OCTA were decreased in patients with pre-perimetric glaucoma^14,16,17^; hence, the utility of OCTA in confirming early glaucomatous changes may increase. OCT and the visual field (VF) test remain the most common methods of evaluating glaucomatous optic nerve damage in clinical practice. However, PERG and OCTA may play an important role in the evaluation of glaucomatous optic nerve damage in the future. Few studies have investigated the correlations between PERG and OCTA parameters, and their role in the diagnosis of glaucoma^18,19^. In addition, there are no studies that have verified the role and correlation of these two parameters in patients with normal-tension glaucoma (NTG). Since it is known that PERG responses are affected by IOP^20,21^, further studies are needed to verify the correlation between PERG and OCTA parameters and diagnostic abilities of these parameters in patients with NTG.

Therefore, in this study, we aimed to explore the relationship between PERG and OCTA parameters for diagnosis in patients with NTG. Further, besides verifying the individual diagnostic abilities of PERG and OCTA parameters, we also evaluated the changes in their diagnostic ability in evaluating glaucomatous functional and structural alterations by combining them with conventional methods.

## Results

In total, 109 patients (49 eyes of 49 normal individuals and 60 eyes of 60 patients with NTG) were included in this study. Table 1 presents the comparison among the groups. Between the normal and the all-glaucoma group, there were significant differences in age, mean deviation (MD), pattern standard deviation (PSD), visual field index (VFI), central sensitivity, ganglion cell-inner plexiform layer (GC-IPL) average thickness, and macular VD. Among the PERG parameters, only the amplitude parameters (N35–P50 and P50–N95) showed significant differences. The sub-group comparison between the normal, early, and moderate to severe groups also revealed significant differences in the variables that were found to be different between the normal and the all-glaucoma group. The results of post hoc analyses are presented in Table 1. Among the PERG parameters, only the amplitude of P50–N95 showed significant differences on sub-group analyses.

**Table 1:** Comparison of demographics and clinical characteristics between normal and patients with NTG.

	Normal (N = 49)	NTG (N = 60)			p^a^	p^b^	p1^c^	p2^d^	p3^e^
		All (N = 60)	Early (N = 29)	Moderate to severe (N = 31)					
Age (years)	48.67 ± 16.01	55.13 ± 15.21	49.38 ± 16.11	60.52 ± 12.28	0.034*	0.002*	0.978	0.003*	0.014*
Sex (M:F)	29:20	34:26	16:13	18:15	0.847	0.624			
CCT (mm)	550.9 ± 49.61	548.8 ± 47.92	554.76 ± 53.97	543.19 ± 43.61	0.820	0.640			
Axial length (mm)	24.64 ± 1.22	25.07 ± 1.77	25.46 ± 1.53	24.71 ± 1.92	0.148	0.059			
IOP (mmHg)	15.47 ± 3.48	15.62 ± 3.03	14.94 ± 3.36	16.34 ± 2.49	0.814	0.234			
MD (dB)	− 0.74 ± 0.84	− 7.28 ± 6.37	− 2.06 ± 1.71	− 12.17 ± 5.08	< 0.001*	< 0.001*	0.132	< 0.001*	< 0.001*
PSD (dB)	1.56 ± 0.42	6.32 ± 4.57	2.47 ± 1.95	9.92 ± 3.13	< 0.001*	< 0.001*	0.124	< 0.001*	< 0.001*
VFI (%)	98.69 ± 2.33	82.82 ± 18.28	96.76 ± 4.17	69.77 ± 16.66	< 0.001*	< 0.001*	0.646	< 0.001*	< 0.001*
Central sensitivity (dB)	32.18 ± 1.11	25.55 ± 6.54	30.22 ± 2.58	21.18 ± 6.1	< 0.001*	< 0.001*	0.056	< 0.001*	< 0.001*
GC-IPL average thickness (μm)	79.82 ± 5.68	66.1 ± 10.35	69.45 ± 7.69	62.97 ± 11.59	< 0.001*	< 0.001*	< 0.001*	< 0.001*	0.008*
Macular vessel density (mm^−1^)	16.67 ± 2.24	12.95 ± 3.76	13.81 ± 3.68	12.15 ± 3.72	< 0.001*	< 0.001*	< 0.001*	< 0.001*	0.103
**Latency (ms)**									
N35	22.68 ± 5.07	22.38 ± 7.58	22.16 ± 5.78	22.59 ± 9.08	0.812	0.942			
P50	50.81 ± 3.83	50.05 ± 5.02	48.93 ± 4.48	51.1 ± 5.34	0.383	0.121			
N95	96.51 ± 8.59	100.6 ± 16.73	98.07 ± 20.64	102.94 ± 11.87	0.125	0.120			
**Amplitude (μV)**									
N35–P50	3.02 ± 0.95	2.21 ± 1.14	2.43 ± 1.26	2.01 ± 0.99	< 0.001*	< 0.001*	0.048*	< 0.001*	0.262
P50–N95	5.19 ± 1.14	3.71 ± 1.43	4.56 ± 1.19	2.91 ± 1.15	< 0.001*	< 0.001*	0.039*	< 0.001*	< 0.001*

Data are presented as mean ± SD or ratio.

*NTG* normal-tension glaucoma, *CCT* central corneal thickness, *GC-IPL* ganglion cell-inner plexiform layer, *IOP* intraocular pressure, *MD* mean deviation, *PSD* pattern standard deviation, *VFI* visual field index.

^a^Independent t-test or chi-square test between the normal and all-glaucoma groups.

^b^Analysis of variance or chi-squared test in the normal, early, and moderate to severe glaucoma groups.

^c–e^p1, p2, and p3 show the results of the post hoc analyses. ^c^p1: normal *vs*. early glaucoma, ^d^p2: normal *vs.* moderate to severe glaucoma, ^e^p3: early glaucoma *vs.* moderate to severe glaucoma.

*p < 0.05.

### Correlation analyses

As the amplitude of P50–N95 was the only significantly different PERG parameter on sub-group analyses, we conducted an age-adjusted correlation analysis between the amplitude of P50–N95 and other parameters for glaucoma evaluation such as IOP, MD, central sensitivity, the amplitude of N35–P50, GC-IPL average thickness, and macular VD (Table 2).

**Table 2:** Age-adjusted correlation analysis between the amplitude of P50–N95 and other parameters for glaucoma evaluation in the normal, early, and moderate to severe NTG groups.

	Normal		Early		Moderate to severe	
	β (95% CI)	p	β (95% CI)	p	β (95% CI)	p
IOP (mmHg)	0.025 (− 0.091 to 0.142)	0.665	− 0.033 (− 0.212 to 0.145)	0.705	− 0.019 (− 0.158 to 0.120)	0.785
MD (dB)	0.214 (− 0.231 to 0.658)	0.338	0.345 (0.113 to 0.578)	0.005*	0.086 (0.002 to 0.170)	0.044*
Central sensitivity (dB)	0.205 (− 0.161 to 0.571)	0.265	0.189 (0.035 to 0.344)	0.018*	0.073 (0.003 to 0.143)	0.043*
Amplitude of N35–P50 (μV)	0.732 (0.418 to 1.047)	< 0.001*	0.339 (− 0.029 to 0.707)	0.039*	0.766 (0.422 to 1.111)	< 0.001*
GC-IPL average thickness (μm)	0.064 (0.002 to 0.125)	0.042*	0.089 (0.040 to 0.137)	0.001*	0.04 (0.005 to 0.076)	0.028*
Macular vessel density (mm^−1^)	0.188 (0.034 to 0.342)	0.018*	0.150 (0.046 to 0.253)	0.006*	0.011 (− 0.109 to 0.130)	0.855

*CI* confidence interval, *IOP* intraocular pressure, *MD* mean deviation, *GC-IPL* ganglion cell-inner plexiform layer.

*p < 0.05.

Table 3 shows the results of age-adjusted correlation analysis between the macular VD and other parameters such as IOP, MD, central sensitivity, the amplitude of N35–P50, the amplitude of P50–N95, and GC-IPL average thickness. The results indicated a significant correlation between the amplitude of P50–N95 and macular VD in the normal and early NTG groups. The amplitude of P50–N95 showed a significant correlation with MD and central sensitivity in the early and moderate to severe NTG groups. However, the macular VD was not significantly correlated with MD and central sensitivity. As for the GC-IPL average thickness, the amplitude of P50–N95 showed a significant correlation in all sub-groups. However, macular VD showed a significant correlation only in the early NTG group.

**Table 3:** Age-adjusted correlation analysis between macular vessel density and other parameters for glaucoma evaluation in the normal, early, and moderate to severe NTG groups.

	Normal		Early		Moderate to severe	
	β (95% CI)	p	β (95% CI)	p	β (95% CI)	p
IOP (mmHg)	− 0.095 (− 0.305 to 0.115)	0.367	0.367 (− 0.216 to 0.950)	0.207	− 0.268 (− 0.706 to 0.170)	0.220
MD (dB)	0.121 (− 0.693 to 0.935)	0.766	0.182 (− 0.726 to 1.089)	0.684	0.096 (− 0.194–0.385)	0.150
Central sensitivity (dB)	− 0.223 (− 0.892 to 0.447)	0.506	0.238 (− 0.332 to 0.808)	0.398	0.209 (− 0.089 to 0.388)	0.171
Amplitude of N35–P50 (μV)	1.039 (0.418 to 1.660)	0.002*	0.634 (− 0.660 to 1.928)	0.323	0.646 (− 0.803 to 2.095)	0.369
Amplitude of P50–N95 (μV)	0.619 (0.113 to 1.126)	0.018*	1.691 (0.520 to 2.862)	0.006*	0.113 (− 1.38 to 1.346)	0.855
GC − IPL average thickness (μm)	0.042 (− 0.074 to 0.158)	0.472	0.189 (0.001 to 0.377)	0.049*	0.042 (− 0.082 to 0.166)	0.486

*CI confidence interval IOP* intraocular pressure, *MD* mean deviation, *GC-IPL* ganglion cell-inner plexiform layer.

*p < 0.05.

### Ability for glaucoma detection

**Figure 1:**
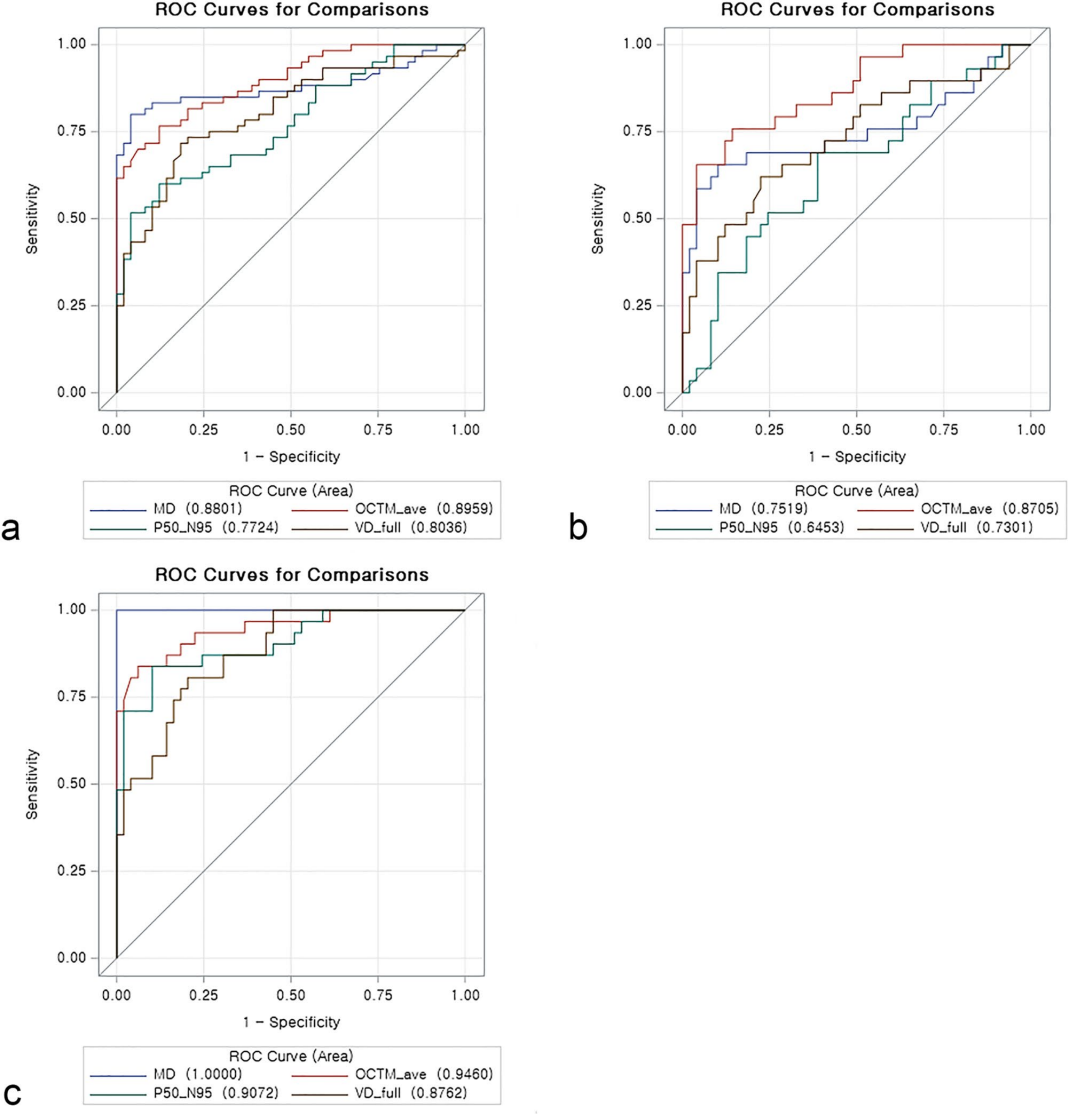
Age-adjusted receiver operating characteristic curves of MD, Ganglion cell-inner plexiform layer average thickness, the amplitude of P50–N95, and macular VD for the detection of glaucoma (**a**), early glaucoma (**b**), and moderate to severe glaucoma (**c**). *MD* mean deviation, *OCTM_ave* Ganglion cell-inner plexiform layer average thickness, *P50–N95* the amplitude of P50–N95, *VD* vessel density.

We investigated the age-adjusted glaucoma detection ability of MD, average GC-IPL thickness, the amplitude of P50–N95, and macular VD. Figure 1 shows the receiver operating characteristic (ROC) curves for each method of glaucoma detection in various sub-groups. MD and average GC-IPL thickness showed a good ability for glaucoma detection in the normal group and the all-glaucoma group, with area under the curve (AUC) values of 0.880 and 0.896, respectively. The amplitude of P50–N95 had the lowest AUC value for glaucoma detection (AUC = 0.772). The order of AUC values for each method (average GC-IPL thickness > MD > macular VD > amplitude of P50–N95) was the same when investigating their ability for early glaucoma detection. Based on the AUC values, significant differences were observed in the ability to detect glaucoma between the amplitude of P50–N95 and MD (p = 0.021), the amplitude of P50–N95 and GC-IPL thickness (p = 0.003), and macular VD and GC-IPL thickness (p = 0.047). While detecting early glaucoma, the AUC value for GC-IPL thickness differed significantly from the amplitude of P50–N95 (p < 0.001) and macular VD (p = 0.034). In the moderate to severe glaucoma group, all methods showed AUC values of > 0.8, and the AUC value for the amplitude of P50–N95 (0.907) was higher than that of the macular VD (0.876). In addition, except for differences in the AUC values between the MD and other variables (MD *vs*. GC-IPL average thickness, p = 0.037; MD *vs*. amplitude of P50-N95, p = 0.008; and MD *vs.* macular VD, p = 0.001), the AUC values of no other parameter for detecting glaucoma differed significantly in the moderate to severe glaucoma group.

By setting the glaucoma detection ability of the MD and average GC-IPL thickness as the reference, we evaluated the additional effects of PERG and OCTA in detecting glaucoma. The diagnostic ability was also calculated by considering the amplitude of P50–N95 and macular VD separately and in combination (Table 4). In all cases, the diagnostic ability was better when compared to the use of the reference method alone, but there were no statistically significant differences. The diagnostic ability for glaucoma was better when the amplitude of P50–N95 was added to the reference than when the macular VD was added. However, the difference was small and non-significant. Since the AUC value of the reference for moderate to severe glaucoma was 1, the results of the additional diagnostic ability for moderate to severe glaucoma are not presented in the Table 4.

**Table 4:** Age-adjusted AUC values for the detection of glaucoma.

	Normal *vs*. all-glaucoma		Normal *vs*. early glaucoma	
	AUC	95% CI	AUC	95% CI
Reference^a^	0.951	0.914–0.988	0.894	0.820–0.969
Reference^a^ + amplitude of P50-N95	0.954	0.919–0.990	0.905	0.836–0.974
Reference^a^ + macular vessel density	0.951	0.912–0.990	0.898	0.822–0.974
All^b^	0.960	0.927–0.993	0.918	0.854–0.983

*AUC* area under the curve, *CI* confidence interval, *MD* mean density.

^a^MD + ganglion cell-inner plexiform layer thickness.

^b^MD + ganglion cell-inner plexiform layer thickness + amplitude of P50-N95+ macular vessel density.

## Discussion

It is known that the amplitude of P50–N95 is related to the function of RGCs^22–24^; hence, the amplitude of P50–N95 is expected to play a clinical role in detecting functional changes in glaucoma. In this study, among the PERG parameters, only the amplitude of P50–N95 showed significant differences between-group comparisons. These results suggest a possible clinical utility of PERG in evaluating glaucomatous damage in patients with NTG. In addition, the amplitude of P50–N95 was significantly correlated with not only MD but central sensitivity in both the early NTG and the moderate to severe NTG group. Thus indicating that PERG may be closely related to the central visual function, as shown in a study that revealed an association between the lower PERG amplitude and parafoveal VF defects^25^. Since the average GC-IPL thickness and amplitude of P50–N95 are associated with the structure and function of the RGC, respectively, a significant correlation between the amplitude of P50–N95 and the average GC-IPL thickness in both early glaucoma and moderate to severe glaucoma groups are reasonable. A similar correlation between the amplitude of P50–N95 and the average GC-IPL thickness was also reported in a previous study^25^.

Since impairment of ocular blood flow is thought to be an important contributing factor for the development and progression of glaucoma^26^, many studies have utilized OCTA, a non-invasive and quantitative technique to visualize microcirculation, for the evaluation of glaucoma status^27–29^. In addition, because RGC bodies and dendrites are mainly located in the macula, it is expected that early glaucomatous damage can be detected in the macular region. Here, on correlation analyses for detecting glaucoma, macular VD showed a significant correlation with the amplitude of P50–N95 in the normal group and the early NTG group and with the average GC-IPL thickness in the early NTG group. The correlation between the amplitude of P50–N95 and the macular VD in the normal and early NTG groups indicate that these parameters can be employed to evaluate the RGC function before severe damage occurs. Whereas, the correlation between the macular VD and the average GC-IPL thickness only in the early NTG group suggests a possible clinical utility of OCTA in assessing the early glaucoma status. However, since the statistical significance was marginal, this result should be interpreted with caution. Further studies involving a larger number of patients with NTG are needed to confirm the correlation between the macular VD and average GC-IPL thickness.

Considering the vascular theory for the pathogenesis of glaucoma, assessment of microvasculature in the macular area using OCTA may act as a sensitive indicator for detecting glaucomatous changes. In this study, macular VD showed a glaucoma detection ability similar to that of MD in eyes with early glaucoma (AUC values for macular VD and MD: 0.730 and 0.752, respectively). Previous studies have demonstrated a range of AUC values for the ability of macular VD (0.562–0.918) in distinguishing eyes with early glaucoma from normal eyes^18,30–33^. Differences in scan size, OCTA specifications, glaucoma severity, and glaucoma type may have contributed to the different outcomes in terms of AUC values; therefore, further studies are warranted to elucidate the exact role of macular VD. However, we observed a similar diagnostic ability between the macular VD and MD in identifying early glaucoma. These results suggest that macular OCTA can be used for the diagnosis of early NTG.

In this study, the amplitude of P50–N95 showed the lowest AUC values in detecting early glaucomatous eyes (AUC = 0.645). North et al. reported an AUC value of 0.787 for PERG in patients with early glaucoma^34^. However, in other studies conducted on patients with early to moderate glaucoma, the AUC value for the amplitude of P50–N90 was 0.34^35^. Jung et al. estimated the glaucoma detection ability of PERG in patients with pre-perimetric and early glaucoma^36^. In their study, the AUC values for the amplitude of P50–N95 in pre-perimetric glaucoma with and without retinal nerve fiber layer (RNFL) defects were 0.779 and 0.618, respectively. In addition, the AUC value for the detection of early glaucoma was 0.847. It could be likely that the type of VF impairment was a major contributor to the various AUC values. As proposed in a previous study conducted on patients with early glaucoma with paracentral scotoma, the location of VF defects should be considered when using PERG for evaluating the glaucoma status^25^. Here, we measured the central sensitivity in all patients. Although there was a difference in the central sensitivity between the normal individuals and the patients with early glaucoma, the difference was not statistically significant (p = 0.056, Table 1). If we would have included a larger proportion of early glaucoma patients with paracentral scotoma, the AUC value for the amplitude of P50–N95 for early detection of glaucoma could have been larger than the current value. However, since we cannot treat patients with glaucoma for a specific type of damage in the real world, our results may reflect the actual clinical scenario. Further studies are needed to identify the specific types of glaucomatous damages in patients with glaucoma, and therefore, PERG may be a more effective test for early diagnosis of glaucoma.

In our study, macular VD showed a higher AUC value (0.804) than the amplitude of P50–N95 (0.772) for the detection of NTG. In the sub-group analysis, macular VD also showed a greater ability to detect early glaucoma than the amplitude of P50–N95 (AUC were 0.730 and 0.645, respectively). However, in the moderate to severe glaucoma group, the amplitude of P50–N95 showed a higher AUC value than the macular VD (0.907 and 0.876, respectively). Furthermore, while evaluating the detection ability of a combination of parameters in patients with early glaucoma, we observed a slightly higher AUC value when the amplitude of P50–N95 (0.905), instead of macular VD (0.898), was added to the reference parameters (MD and average thickness of GC-IPL). The results suggested that the role of OCTA and PERG parameters in the detection of glaucoma may vary according to its severity. However, in comparison to the reference parameters, the improvement in diagnostic ability on adding the parameters, such as the amplitude of P50–N95 and/or macular VD, was limited. Therefore, further studies are necessary to determine the conditions where PERG and OCTA parameters may improve the diagnostic power for glaucoma.

Few studies have compared the diagnostic ability of PERG and OCTA parameters in patients with glaucoma. Kurysheva et al.^18^ compared the diagnostic abilities of various parameters, including those related to PERG and OCTA, in patients with primary open-angle glaucoma. In their study, AUC values for whole image en face VD and the amplitude of P50–N95 for detecting early glaucoma were 0.800 and 0.893, respectively. Regarding the diagnostic ability of macular VD and the amplitude of P50–N95 in patients with early glaucoma, their findings were consistent with the results of our study. However, in their study, the diagnostic ability of the thickness of the ganglion cell complex and MD were lower than that of macular VD and the amplitude of P50–N95); the results differed from our findings. In comparison to our study, they measured VD in a different area using a different model of OCTA and conducted ophthalmological examinations after discontinuing topical anti-glaucoma medication; hence, their study findings cannot be directly compared with our results. It is well-known that PERG responses are affected by IOP^20,21,37^. Therefore, changes in IOP after discontinuing topical anti-glaucoma medication may be a major confounding factor when using PERG for glaucoma evaluation. The influence of IOP on PERG can vary depending on whether the research was conducted in glaucoma patients with high or low IOP. In this study, we only included patients with NTG. The pre-treatment IOP was not significantly different between the normal individuals and the patients with NTG. In addition, pre-treatment IOP was not significantly correlated with the amplitude of P50–N95 on correlation analyses. These results suggest that PERG can be useful in assessing the RGC function in NTG patients, and the correlation between PERG and OCTA parameters exists irrespective of the IOP status, highlighting the possible clinical utility of PERG and OCTA parameters for evaluating glaucomatous optic nerve damage in patients with NTG.

The main limitation of our study was its retrospective study design and small sample size. In addition, because the severity of glaucoma in all patients was evaluated by the VF test, it was not possible to evaluate glaucomatous changes that preceded the perimetric deterioration. However, perimetry is a standard method for detecting glaucomatous functional damage, and, in general, the severity of glaucoma is classified according to the VF test results; therefore, this limitation is unavoidable until a new method is developed for detecting glaucomatous damage. Here, we only evaluated OCT and OCTA parameters in the macular region. However, considering that PERG can identify the function of RGCs in the macular area, the use of only the macular parameters of OCT and OCTA would serve as a condition to obtain highly reliable results in identifying the association with the PERG parameters. Lastly, although we calculated the central sensitivity using the 24-2 VF test to measure functional damage in the macular area, central VF damage was not evaluated by the 10-2 VF test. Further studies involving a 10-2 VF testing in patients with NTG are required for assessing the usefulness of PERG and OCTA in evaluating glaucomatous changes. Despite these limitations, we could confirm the relationship between PERG and OCTA parameters in patients with NTG and their potential as an adjuvant method for detecting NTG.

In conclusion, our study demonstrated significant correlations between PERG and OCTA parameters in patients with early NTG. The detection ability of macular VD for early glaucoma was similar to that of MD. In addition, there was more improvement in the detection ability on adding the amplitude of P50–N95, compared to macular VD, to the conventional reference methods comprising MD and average thickness of GC-IPL. The results indicate that PERG and OCTA parameters may play an important role in detecting early NTG. Further, this study demonstrates the importance of finding conditions under which the diagnostic abilities of PERG and OCTA parameters can be optimized for glaucoma. The diagnostic ability of macular VD was higher than that of the amplitude of P50–N95 in patients with early NTG, and a reverse pattern was observed in patients with moderate to severe NTG, indicating that the roles of PERG and OCTA parameters may vary depending on the severity of the glaucomatous damage.

## Methods

### Patients

In this retrospective study, we reviewed the medical records of patients that were examined at the Glaucoma Clinic of the Department of Ophthalmology of Severance Hospital, Yonsei University School of Medicine, Seoul, Korea between November 2017 and October 2019. The study was approved by the Institutional Review Board of Yonsei University Severance Hospital, Seoul, Korea (1-2019-0007) and was conducted according to the tenets of the Declaration of Helsinki. The requirement for informed consent was waived.

The enrolled patients underwent a comprehensive ophthalmologic examination routinely performed at our clinic for evaluating the optic nerve status. The examination included the Snellen’s eye chart examination for best-corrected visual acuity, Goldmann applanation tonometry for IOP, and slit-lamp bio-microscopy. RNFL defects and optic disc morphology were evaluated using a + 90-diopter (D) lens, red-free photography (VISUCAM 200; Carl Zeiss Meditec AG, Jena, Germany), and spectral-domain OCT (Cirrus 5000 HD OCT; Carl Zeiss Meditec AG). The VF was analyzed using standard automated perimetry (SAP) (Humphrey Field Analyzer; 24-2 Swedish Interactive Threshold Algorithm; Carl Zeiss Meditec AG) to evaluate the visual function. All examinations, including PERG and OCTA, were performed on the same day. The patients who met the following criteria were included: best-correct visual acuity of 20/25 or better; the spherical equivalent of < 5 D; cylindrical refractive error of < 2 D; axial length of < 25.5 mm; any type of cataract with a severity grade of < 3 (Lens Opacities Classification System III)^38^; no use of systemic medications; and no pre-existing retinal or optic nerve disorders, systemic or ocular conditions affecting visual function, or previous history of any intraocular surgery. The patients with a medical history of anti-glaucoma medication usage, before being diagnosed at our clinic, or an IOP of > 18 mmHg during the follow-up period were excluded from the study.

By reviewing the results of these ophthalmological examinations and medical records during the follow-up period, we reassessed whether the patients had NTG. In this study, NTG was defined as glaucomatous changes in the optic disc and related defects in the RNFL and VF, accompanied by an open angle, with a maximum untreated IOP of < 18 mmHg on three repeated measurements obtained at different times on separate visits^38^. At least two of Anderson’s criteria (false-positive errors < 15%, false-negative errors < 15%, and fixation loss < 20%) had to be satisfied for a VF defect to be considered glaucomatous. According to Hodapp–Parrish–Anderson’s criteria, patients with glaucoma were classified into early or moderate to severe groups^39^. Two glaucoma specialists (SYL and HWB) reviewed the medical records to re-evaluate the diagnosis of NTG. In cases of disagreement, another glaucoma specialist (CYK) determined whether NTG was present. The eye that had more severe glaucoma was selected. If both eyes showed normal or the same degree of glaucoma severity, the study eye was randomly selected.

### Parameters for OCT and VF

Macular GC-IPL thickness was evaluated using a Cirrus HD OCT 512 × 128 macular cube scan over 6 × 6 mm^2^ macular areas centered at the fovea. The elliptical annulus consisted of an inner and outer vertical diameter of 1 mm and 4 mm, respectively, and an inner and outer horizontal diameter of 1.2 mm and 4.8 mm, respectively. Among the various parameters (such as average, minimum, and six sectoral thicknesses), the average macular GC-IPL thickness was used for analyses.

The MD, PSD, and VFI measured by SAP were used as VF parameters. Additionally, to evaluate the visual function of the central region, we used the central sensitivity, which was calculated by averaging the sensitivity for 12 central cluster points that were considered to match topographically to the 4.8 mm retinal area centered at the fovea, as described in previous studies^40,41^.

### Parameters for OCTA and PERG

**Figure 2:**
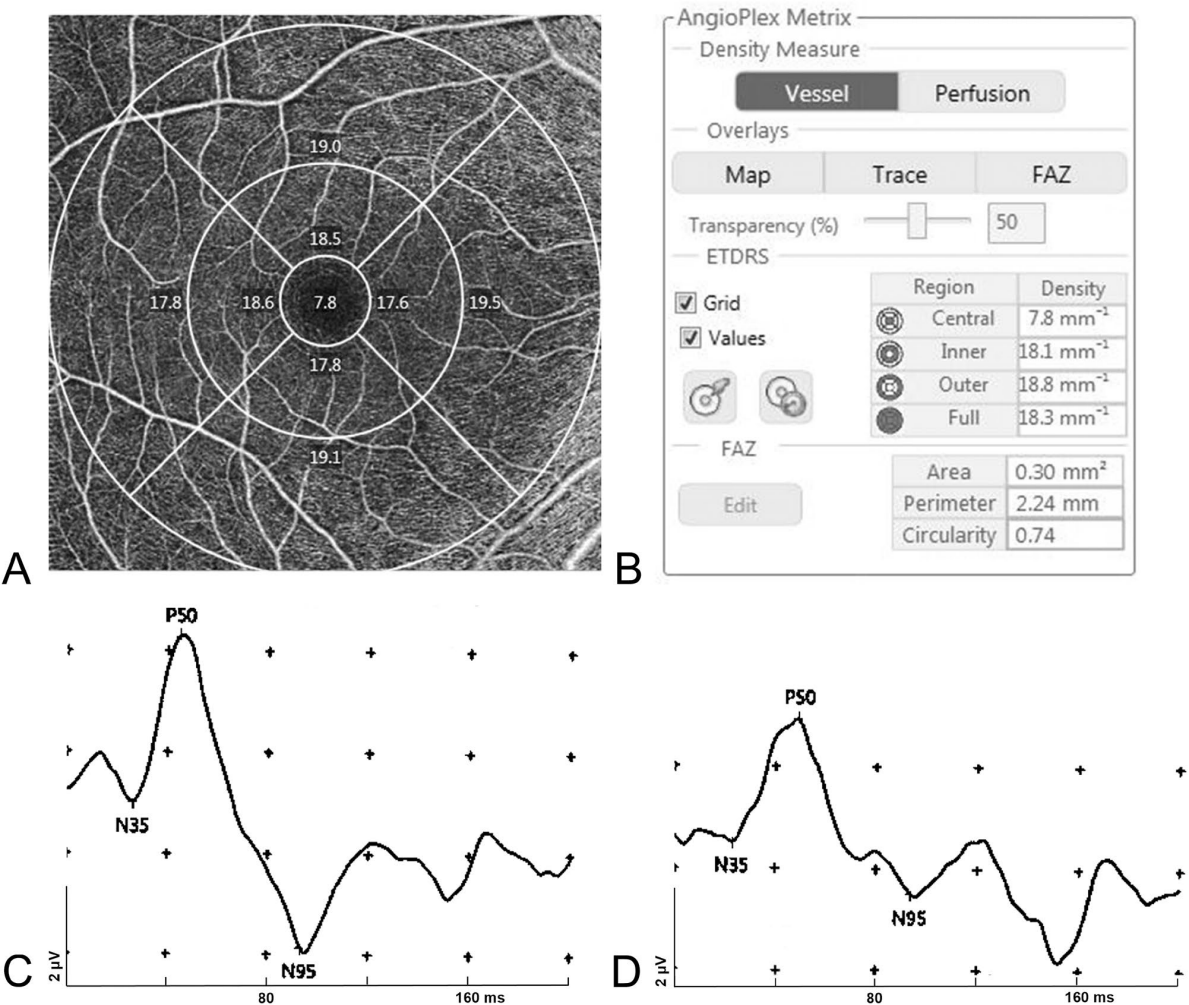
Measurement sectors for the vessel density of optical coherence tomography angiography (**a,b**). A representative waveform of the pattern electroretinogram in a glaucoma suspect (**c**) and a patient with normal-tension glaucoma (**d**).

All participants underwent Angioplex OCT angiography on the Cirrus 5000 HD OCT system with a 6 × 6 mm^2^ area pattern centered on the fovea. This examination was performed after pupil dilation. Participants whose images had artifacts resulting in segmentation errors or signal strength of < 7 were excluded. The VD of the superficial vascular plexus (from the internal limiting membrane to the inner plexiform layer) was calculated using a built-in software at all nine Early Treatment Diabetic Retinopathy Study sectors. The VD was assessed in both four inner and outer sectors and all sectors. Among these parameters, the VD, calculated for all sectors, was used for analyses (Fig. 2a,b).

PERGs were measured by a Neuro-ERG system (Neurosoft, Ivanovo, Russia) using a previously described method^36,42^, that has been reported to have an excellent reproducibility^25^. The examination was conducted with full optical correction in a room with a constant background illumination of 50 lx. The patients were made to sit, and two skin electrodes and two ground electrodes were placed on their lower lids and earlobes, respectively. A black and white checkerboard pattern with a check size of 1.81° was presented on a 24-inch LCD monitor (corresponding to 48° × 33° of the visual angle in the VF) held at a distance of 60 cm. The delay time of the LCD monitor was set to 15 ms according to the manufacturer’s recommendation to prevent the effect of the luminance artifact. The mean luminance of the checkerboard pattern was 100 cd/m^2^. The fixation point was a red dot at the center of the screen. The stimulus–response, an average of ≥ 100 recorded results, was band-pass filtered (1–50 Hz) with a sampling rate of 10,000 Hz. The responses were simultaneously measured in both eyes under non-dilated condition.

In this study, we used the following PERG parameters^6,43,44^: (1) amplitude of N35–P50: the first positive value, calculated as the voltage difference between the first prominent negative value and the first prominent positive value, (2) amplitude of P50–N95: the first negative value, calculated as the voltage difference between the maximum peak and the subsequent trough, and (3) the implicit time (latency) for N35, P50, and N95: the time from the start of the checkerboard reversal to the appearance of a peak for each component.

Representative PERG results for a normal individual and a patient with NTG are presented in Fig. 2c and d, respectively.

### Statistical analyses

Statistical analyses were performed using SAS version 9.4 software (SAS Institute Inc., Cary, NC, USA). Between-group comparisons were performed using the independent two-sample t-test for continuous variables, and the chi-square test and analysis of variance for categorical variables. Post hoc analysis was carried out using Scheffe’s method. The age-adjusted correlation was assessed using regression analysis. The ROC curve was calculated to evaluate the diagnostic power of glaucoma. To compare diagnostic ability, AUCs were compared using Delong’s method. Statistical significance was defined as p < 0.05.

## Data Availability

The datasets generated during and/or analyzed during the current study are available from the corresponding author on reasonable request.
